# Effect of Exercise Prior to Sedentary Behavior on Vascular Health Parameters: A Systematic Review and Meta-Analysis of Crossover Trials

**DOI:** 10.1186/s40798-024-00734-4

**Published:** 2024-06-09

**Authors:** Francisco Javier Soto-Rodríguez, Alicia Peris Moya, Carolina Javiera Bobadilla-Agouborde, José Manuel Pérez-Mármol

**Affiliations:** 1 Departamento de Ciencias de la Rehabilitación, Facultad de Medicina, Universidad de La Frontera, Temuco, Chile; 2 Facultad de Ciencias de la Salud, Carrera de Kinesiología, Universidad Autónoma de Chile, Temuco, Chile; 3 Departamento de Fisioterapia, Facultad de Ciencias de la Salud, Universidad de Granada, Granada, Spain; 4 Departamento de Procesos Terapéuticos, Facultad de Ciencias de la Salud, Universidad Católica de Temuco, Temuco, Chile; 5 Instituto de Investigación Biosanitaria (ibs.GRANADA), Granada, Spain

**Keywords:** Sedentary behavior, Vascular health, Endothelial function, Exercise

## Abstract

**Background:**

Sedentary behavior has been shown to negatively affect parameters of endothelial function and central hemodynamics, both of which are closely associated with vascular health. Exercise prior to sedentary behavior has demonstrated potential as a preventive strategy to mitigate these detrimental effects. To evaluate the impact of exercise prior to sedentary behavior on vascular health parameters in the adult population, a systematic review and meta-analysis were conducted, synthesizing the available body of knowledge.

**Methods:**

A literature search was carried out in 6 databases. For each outcome, standard error and mean difference or standardized mean difference were calculated, as appropriate. An analysis was performed using a random effects model with a 95% confidence interval, using the inverse variance statistical method. Risk of bias assessment was performed using ROB2 and considerations for crossover trials. The quality of evidence was assessed using the GRADE system.

**Results:**

Exercise performed prior to prolonged sedentary behavior resulted in increased flow-mediated vasodilation at the first and third hours of sedentary time, compared with the control condition of sedentary behavior without prior exercise [MD: 1.51% (95% CI: 0.57 to 2.45) and MD: 1.36% (95% CI: 0.56 to 2.16), respectively]. Moreover, prior exercise led to increased shear rate at the first and third hours of sedentary time [MD: 7.70 s^^−1^ (95% CI: 0.79 to 14.61) and MD: 5.21 s^^−1^ (95% CI: 1.77 to 8.43), respectively]. Furthermore, it increased blood flow at the third hour [SMD: 0.40 (95%CI: 0.07 to 0.72)], compared with the control condition of prolonged sedentary behavior without prior exercise. Regarding hemodynamic parameters, exercise prior to prolonged sedentary behavior decreased mean arterial pressure during the first and third hours of sedentary behavior [MD: -1.94 mmHg (95% CI: -2.77 to -1.11) and MD: -1.90 mmHg (95% CI: -3.27 to -0.53), respectively], and an increase in heart rate during the first hour [MD: 4.38 beats per minute (95%CI: 2.78 to 5.98)] compared with the control condition of prolonged sedentary behavior without prior exercise.

**Conclusions:**

The findings of this research suggest that prior exercise may prevent the impairment of vascular health parameters caused by sedentary behavior. However, the quality of the evidence was estimated as moderate. Therefore, further experimental studies and high-quality clinical trials are needed in this field to strengthen the results and conclusions drawn.

**PROSPERO registration number:**

CRD42023393686.

## Key Points

- Engaging in exercise prior to sedentary behavior seems to exert a preventive effect on vascular health parameters, both after one hour and three hours of being sedentary.
- Exercise prior to sedentary behavior could be an effective strategy in preventing endothelial dysfunction.
- The preventive impact of exercise prior to sedentary behavior may be comparable in magnitude to the benefits derived from interrupting prolonged sitting.

## Background

Levels of sedentary behavior (SB) in Western populations have increased dramatically, a trend attributed to rapid technological advances and the economic, social, and environmental transformations experienced in recent decades [1]. The Sedentary Behavior Research Network defines SB as a state of wakefulness characterized by energy expenditure ≤ 1.5 metabolic equivalents (METs) while sitting, reclining, or lying down [2]. In this context, it has been observed that daily exposure to this behavior could encompass between half and three-quarters of the time that individuals remain awake [3, 4]. Furthermore, studies in North American populations have found that exposure times to SB range between 9.8 [5] and 11 h per day [6].

SB is considered a determinant factor in the development of cardiovascular and metabolic diseases [1] and is closely associated with an increase in cardiovascular and all-cause mortality [7, 8]. These adverse effects could be partly explained by the hemodynamic alterations induced by this behavior, leading to disruptions in vascular homeostasis [9, 10]. SB has been shown to have a negative impact on vascular health and is linked to the development of endothelial dysfunction, a crucial prognostic indicator for overall cardiovascular disease risk [11, 12]. In line with this, a 1% decrease in endothelial function has been estimated to increase cardiovascular risk by 13% [13].

Endothelial dysfunction refers to a subclinical condition, which is characterized by an imbalance between the vasodilator and vasoconstrictor factors of the endothelium [14]. This condition implies the alteration of other properties linked to this vascular layer such as fibrinolytic, anti-inflammatory, antiplatelet and anticoagulant capacity [15]. Currently, it is recognized that endothelial dysfunction precedes the morphological changes of atherogenesis and exacerbates the adverse effects of atherosclerosis [16]. A change in the endothelial cells function is related to a lower synthesis of nitric oxide (NO), greater oxidative stress, lipid deposits, and an increased risk of coronary thrombosis, also being considered as an early stage of heart failure [17, 18].

Although all forms of SB have the potential to initiate hemodynamic alterations responsible for endothelial dysfunction, prolonged sitting time has been the main focus of interest and study. Prolonged sitting has been shown to induce acute endothelial dysfunction due to decreased blood flow (BF) in large vessels, leading to reduced shear stress (SS) [19, 20]. These alterations impact the mechanisms involved in nitric oxide (NO) synthesis, resulting in a reduction in endothelial vasodilator capacity, represented by a decrease in the percentage of flow-mediated dilation (%FMD) [21]. In agreement with this, a trial involving 11 healthy men reported that 6 h of prolonged sitting time affects lower extremity endothelial function, causing a reduction in %FMD, SS, and BF [22]. Similarly, Credeur et al. [23] found that 3 h of sedentary time decreased %FMD and SS in a sample of 20 overweight adults. Other trials have indicated that the negative effects of prolonged sitting on endothelial function can manifest as early as the first hour of sedentary exposure [24–26].

Considering this background, SB could also negatively impact central hemodynamic parameters, such as blood pressure (BP) and heart rate (HR), resulting in an increase in both. This phenomenon could be explained by the increase in sympathetic activity driven by the reduction in pressure on arterial baroreceptors due to venous pooling caused by SB [11, 27]. O’Brien et al. [28] found that 3 h of prolonged sitting caused increases in both BP and HR. These findings align with those reported by Kowalsky et al. [29], who observed similar effects after 4 h of this behavior. Given these literature reports, it seems relevant to investigate the effectiveness of existing strategies aimed at mitigating the effects of SB on vascular health.

Recent studies have demonstrated that engaging in exercise can counteract the effects of SB on endothelial function [30–34]. Along these lines, strategies to interrupt prolonged sitting through activities such as walking or stair climbing [24, 35, 36] or simple resistance exercises (i.e., body weight-resisted exercises) [25] have been found to prevent the reduction in %FMD, SS, and BF. Furthermore, recent trials have investigated the potential preventive effect of performing exercise prior to a session of SB (e.g., prior exercise to prolonged sitting) on endothelial dysfunction. Ballard et al. [30] reported that 45 min of aerobic exercise on a treadmill (corresponding to 65% VO2max) prior to 3 h of prolonged sitting prevented the decrease in %FMD. Similarly, other prior exercise strategies, such as cycling at moderate intensity, appear to improve %FMD, SS, and BF compared to a control condition (no prior exercise) [32]. In contrast to these reports, a study conducted by Weston et al. [37] found changes in endothelial function only with high-intensity interval prior exercise (prior to SB), not with moderate-intensity exercise. This mode of exercise before exposure to SB could also have a favorable effect on central hemodynamic parameters such as BP and resting HR [38]. However, due to the heterogeneity of the findings in the existing literature, a clear consensus has yet to be reached.

To the best of our knowledge, there are no systematic reviews or meta-analyses evaluating the effect of exercise prior to SB on vascular health parameters. Thus, and in response to the lack of consensus, the objective of this study is to assess the existing body of knowledge regarding the effects of exercise prior to SB on vascular health parameters in the adult population. The study hypothesis is that this type of exercise strategy prevents the impairment of vascular function and central hemodynamic parameters caused by a prolonged session of SB.

## Methods

### Design

This current systematic review and meta-analysis has been conducted following the general methods of Cochrane reviews. The Preferred Reporting Items for Systematic Reviews and Meta-Analyses (PRISMA) have also been followed [39]. The review protocol was prospectively registered in PROSPERO on February 5, 2023, under registration ID: CRD42023393686.

### Identification and Selection of Studies

Firstly, database searches were performed in Cochrane Plus, Cochrane Library, PROSPERO, and the ProQuest Platform. Databases of gray literature were also consulted such as TESEO (official Spanish database for PhD theses), ProQuest Dissertations and Theses Global, OATD (Open Access Theses and Dissertations) and Google Scholar. No studies pursuing the same aims as the present study were found.

Subsequently, a database search for original/primary studies was performed using PubMed, Central (the Cochrane Central Register of Controlled Trials), Scopus, Web of Science, SPORTDiscus, and CINAHL. The search in these databases was independently performed by two researchers. For this process, the following keywords were used: “exercise”, “physical exercise”, “prior exercise”, “resistance exercise”, “aerobic exercise”, “sedent*”, “sedentary behavior”, “sedentary time”, “sitting time”, “endothelial function”, “endothelial dysfunction”, “vascular function”, “flow-mediated dilation”. The search equation is provided in Supplementary Material #1.

For the management of the selection process, the Rayyan online tool (along with its mobile application) was employed to facilitate the selection of articles based on titles, abstracts, and full text. This was conducted independently by two researchers and any disagreements were resolved through further discussion and consensus with the research team. Eligibility criteria were considered for the careful selection of articles.

The study eligibility criteria were as follows:

- Study design: Parallel or crossover clinical trials.
- Population: Non-exercise-trained healthy adults (over 18 years old) exposed to SB.
- Intervention: Primary studies that include interventions based on physical exercise prior to SB, or studies whose interventions are focused on improving endothelial function or reversing the impact of SB on endothelial function. Articles needed to include measurement of the vascular response of the endothelium of large arteries.
- Comparator: Primary studies including a control group as comparator of the intervention. The participants from this group do not perform any type of exercise or movements prior to SB, that is, these participants maintain uninterrupted SB throughout since the onset to the end of the study participation.
- Outcomes measures: Studies that evaluate the effect of interventions on peripheral vascular parameters (%FMD, SS, and BF) and central hemodynamic parameters (HR and BP).

Exclusion criteria for this study included:

- Interruption strategies to manage endothelial dysfunction were excluded from the study.
- Primary studies including participants with conditions that required them to maintain an uninterrupted sitting position (i.e., wheelchair users, bedridden individuals, older adults with mobility impairments, and similar conditions).

### Data Extraction

The data extraction was performed in a data spreadsheet by two independent researchers and subsequently a third researcher reviewed that all extracted data were consistent. The following information was extracted from the selected studies: (1) first author’s name, (2) year of publication, (3) study design, (4) sample size, (5) participant characteristics (i.e., age, sex, body mass index), (6) technique for assessing vascular and central hemodynamic parameters, (7) intervention characteristics, and (8) outcome measures. The mean and standard deviation of the intervention and control conditions related to SB were also extracted for each of the outcome measures, that is, %FMD, shear rate (SR) (as a measure of SS), BF, BP, and HR, along with the sample size of the groups. These data were extracted with the purpose of generating mean effect size and confidence intervals for subsequent meta-analysis. In cases where the quantitative data provided in the article were incomplete, the authors of the primary studies were contacted via email and/or ResearchGate to request the missing data.

### Statistical Analysis

For statistical analysis and development of this meta-analysis, Review Manager (RevMan) 5.4 software [40] was used. Since the primary studies included in this meta-analysis have a crossover design, the methodological recommendations of Elbourne et al. [41] were followed to avoid a unit-of-analysis error for the estimation of the mean effect size. Mean difference, standardized mean difference, and standard error calculations were performed on a Microsoft Excel spreadsheet and then entered into RevMan as a generic inverse variance variable. Studies that did not provide complete data and for which calculations were not possible through other means were excluded from the meta-analysis, but not from the systematic review. A random effects model was used to provide mean difference and standardized mean difference with a 95% confidence interval, using inverse variance as the statistical method. Mean difference was used for the analysis of variables that were measured on the same scale in the studies, such as FMD, SR, BP, and HR. Standardized mean difference was used to evaluate the magnitude of the effect in situations where the measurement scale was different, such as in the case of SR_AUC_ and BF. An effect size of < 0.2 was considered trivial, between 0.2 and 0.49 small, 0.50 and 0.79 moderate, and ≥ 0.8 large [42]. Sensitivity analyses were performed to evaluate the robustness of the results using the sequential elimination method. The influence of each study on the overall results of the meta-analysis was assessed. Statistical heterogeneity was evaluated using I^2^, where values < 25%, 25 − 75%, and > 75% indicate low, moderate, and high heterogeneity, respectively [43].

### Risk of Bias Assessment and Methodological Quality

Since the included studies were crossover trials, the latest version of the RoB 2 tool for crossover trials [44] was used to assess the risk of bias. This tool enables the evaluation of six types of bias: (1) arising from the randomization process, (2) arising from period and carryover effects, (3) due to deviations from the intended intervention, (4) due to missing outcome data, (5) in measurement of the outcome, and (6) in selection of the reported result. The assessment of bias risk is reported as a summary table for each article, as well as the percentage of bias grouped for all included studies. Two researchers conducted the risk of bias assessment in parallel and discrepancies were resolved by consensus after discussion.

To evaluate the quality of evidence and confidence in the estimation of effects, the GRADE system (Grading of Recommendations, Assessment, Development and Evaluation) [45] and corresponding web-based software GRADEpro GDT [46] were used. This system is based on three aspects that allow the establishment of confidence in the effect of an intervention. These aspects refer to: (1) study design, (2) factors that decrease confidence, and (3) factors that increase confidence. Based on these, the quality of evidence is classified as high, moderate, low, or very low, considering the design of the primary studies (randomized studies are considered high quality, while observational studies are considered low quality). Furthermore, this quality category can be modified in the presence of certain factors such as risk of bias, design limitations, lack of directionality, unexplained heterogeneity, imprecision of results, or likelihood of publication bias. In this sense, high or moderate quality evidence reflects confidence regarding the effects of the intervention, while low or very low quality evidence reflects uncertainty [45]. To determine the quality of evidence regarding exercise prior to SB on vascular health parameters, five outcomes were evaluated, with two being identified as critical (%FMD and BP) and three considered important (SR, BF, and HR).

## Results

### Literature Search and Study Characteristics

**Fig. 1:**
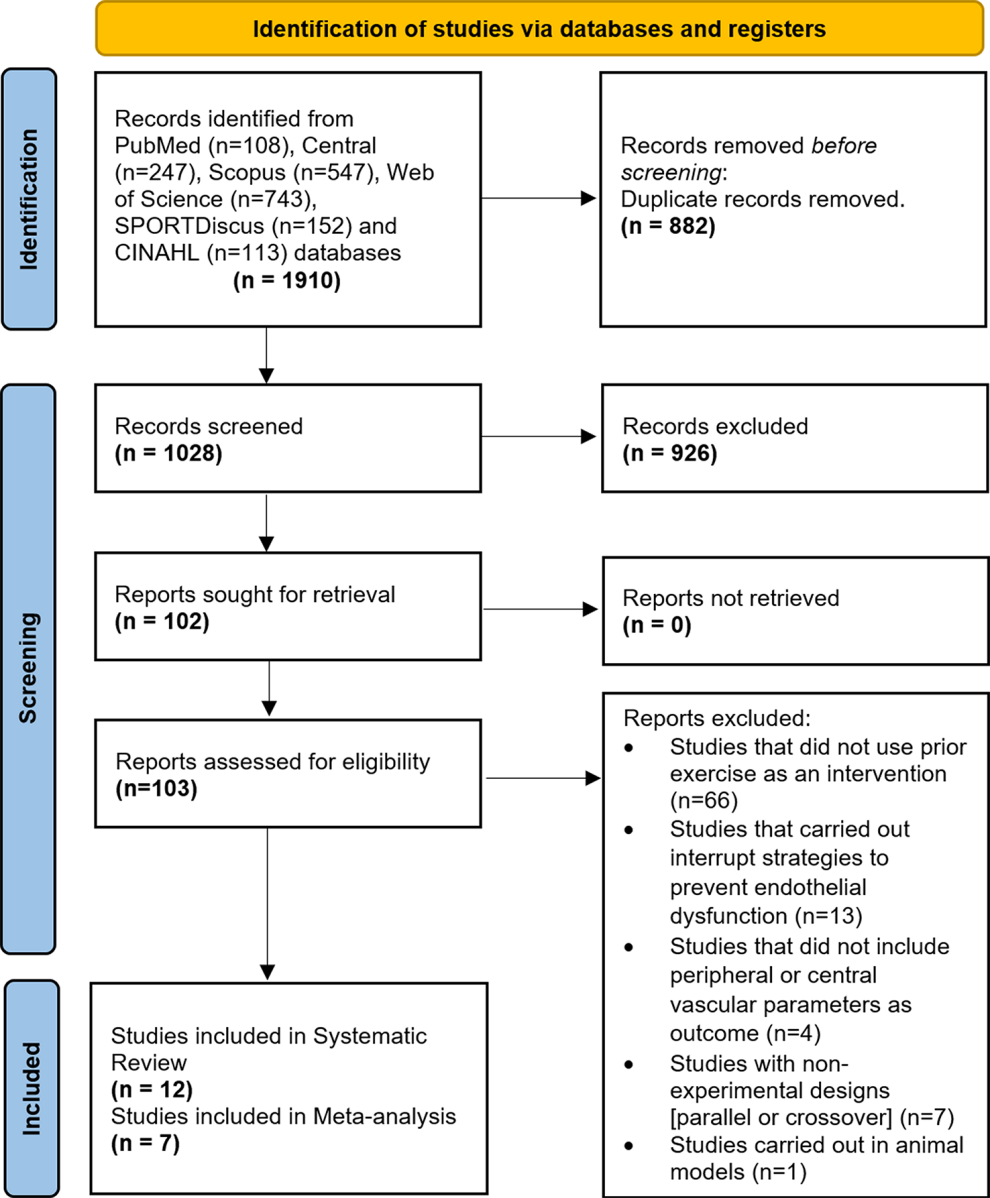
PRISMA flow diagram

The systematic literature search identified 1910 records. The flow diagram is depicted in Fig. 1. Of these records, 743 were retrieved through Web of Science, 547 through Scopus, 247 through Cochrane Central Register of Controlled Trials, 152 through SPORTDiscus, 113 through CINAHL, and 108 articles were retrieved from PubMed. After removing duplicates and conducting the screening process, 12 articles were included in the qualitative analysis. Regarding vascular function parameters, four studies reported results of prior exercise on FMD [30, 32, 37, 47] four on SR [30, 32, 37, 47], and two on BF [31, 32]. Regarding central hemodynamic parameters, six studies reported results on HR [38, 48–52], and ten studies on BP [30, 32, 37, 38, 48–53].

For the quantitative analysis, seven articles were included. Four articles provided data on vascular function variables, with three studies contributing data on the effects on FMD [30, 32, 37] and SR [30, 32, 37], and two studies on BF [31, 32]. For hemodynamic parameters, data from six articles were used, with three studies examining the effects on HR [38, 48, 51] and five studies on BP [30, 32, 37, 38, 51]. All studies employed a randomized crossover controlled trial design, with sample sizes ranging from 10 [31, 37, 52] to 67 individuals [49]. The total number of subjects participating in the studies was 237, with 64% being male, and ages ranging from 19 to 77 years.

### Exercise Interventions Prior to Sedentary Behavior

The studies used experimental intervention strategies based on prior exercise to SB through different modalities such as: cycling on a cycle ergometer at a light-intensity [48], moderate-intensity strategies such as walking on a treadmill [30, 38, 49, 50] or cycling [32, 37, 51, 52], and high-intensity strategies practiced in an interval modality [31, 37, 47, 53]. These interventions involved prior exercise application lasting between 10 [38] and 45 min [30, 32, 48]. The characteristics of the included studies and intervention details are presented in Table 1.

**Table 1:** Characteristics of the included studies

Reference	Study design	Sample Size	Sex	Age (± SD)	IMC (kg/m^2^)	Assessment of Vascular Function parameters	Assessment hemodynamic parameters	Intervention details	Effect on Vascular Function parameters	Effect on hemodynamic parameters
Weston et al. [37]	Randomized cross-over trial	10	6 women 4 men	22.7 ± 3.5	23.3 ± 2.1	Assessed with high-resolution ultrasonography on the brachial artery, which allowed the calculation of %FMD and SR.	N/E	Participants completed 4 interventions. (1) Remain seated and at rest throughout the intervention period [Control Trial], (2) 30 min of cycling at 60% VO2max [moderate intensity], (3) 30 min of high intensity interval exercise at 75% intervals VO2max [HIIE1] and, (4) 30 min of high intensity interval exercise at 90% VO2max [HIIE2]. After the interventions, the participants remained at rest for 3 h.	**FMD**_**1h**_ (first hour): positive effect of HIIE1 and HHIE2 compared to control trial. **FMD**_**3h**_ (third hour): no effect of any intervention compared to control trial. **SR**_**AUC**_: no effect of any intervention compared to control trial at 1–3 h	**BP**: no effect of any intervention compared to control trial 1–3 h
Fecchio et al. [48]	Randomized cross-over trial	30	6 women 24 men	42 ± 11	30.1 ± 5.1	N/E	BP was assessed with an auscultatory technique in the dominant arm and a mercury sphygmomanometer. Electrocardiography was used for HR.	The participants underwent four experimental sessions, consisting of two exercise and two control sessions performed in a randomized order. Experimental sessions were divided in two blocks: test and retest. (1) In exercise sessions, they exercised for 45 min on a cycle ergometer at 50% of VO2 peak and, (2) in the control sessions, they stayed seated on the cycle ergometer for 45 min without pedaling, After the interventions, the patients remained seated for 55 min.	N/E	**HR**: HR increases with exercise compared to control trial at 1 h of sedentary behavior. **BP**: positive effect of exercise compared to control trial at 1 h of sedentary behavior.
Freire et al. [53]	Randomized cross-over trial	25	15 women 10 men	24.4 ± 3.8	26.1 ± 3.4	N/E	BP was assessed with an oscillometry device.	Participants underwent 3 different 10-hour experimental interventions. (1) 10 h of sedentary behavior [prolonged sitting], (2) Exercise prior to sedentary behavior [8.3 h], characterized by a single session of Low-Volume High-Intensity Interval Exercise [LV-HIIE] 75 min after the breakfast (5 min of warm-up at 4 km/h, 10 × 1 min at 100% of peak treadmill velocity with 1 min of passive recovery, and 2 min of cooldown at 4 km/h) and, (3) 10 h of interrupted sitting time with 5 min walks at habitual gait speed every 20 min reaching 10,000 steps [*This intervention has not been considered in the analysis*].	N/E	**BP**: no effect of LV-HIIE compared to control trial (10 h of sedentary behavior) at any time.
Perdomo et al. [38]	Randomized cross-over trial	15	13 women 2 men	45.4 ± 8.9	26.4 ± 4.2	N/E	Central vascular parameters at rest, BP and HR were measured together with an automatic blood pressure monitor. During the intervention, HR was measured with a continuous chest pulsometer.	Participants underwent three experimental sessions. (1) a 10-min exercise bout, (2) a 30-min exercise bout [Both exercise interventions consisted of walking on a treadmill at 70-75% of age-predicted maximum heart rate. Subsequently, the participants remained in the supine position for 1 h] and (3) A control session characterized by 30 min of being in a seated position followed by 1 h in a supine position.	N/E	**HR**: HR increases with exercise compared to control trial at 1 h of sedentary behavior. **BP**: positive effect of exercise trials compared to control trial at 1 h of sedentary behavior.
Garten et al. [31]	Randomized cross-over trial	10	10 men	24 ± 1	26 ± 1	Assessed with Doppler ultrasonography over the common femoral artery in order to measure changes in BF.	N/E	Participants completed 2 counterbalanced experimental interventions performed on separate days involving (1) a sitting only session and (2) a sitting session with a high-intensity interval aerobic exercise [HIIE] session performed immediately before. The HIIE bout lasted for 28 min and consisted of 4 bouts of 4 min of cycling exercise performed at a workload corresponding with 85 to 95% HR peak followed by 3 min of cycling performed at a workload corresponding to 50% HRpeak.	**BF**: positive effect of HIIE compared to sitting session trial. *Regarding the Peak change in Leg blood flow.* No effect on resting BF	N/E
Wheeler et al. [49]	Randomized cross-over trial	67	35 women 32 men	67±7	31.2 ± 4.1	N/E	Resting blood pressure and heart rate were measured by an automatic oscillometric device.	Participants completed 3 laboratory trial conditions. (1) uninterrupted sitting [8 h, control], (2) exercise prior to sitting: sitting [1 h], then moderate-intensity treadmill walking for 30 min followed by uninterrupted sitting [6.5 h] and (3) exercise + breaks: sitting [1 h], then moderate-intensity treadmill walking for 30 min followed by sitting [6.5 h] interrupted every 30 min with 3 min of light-intensity treadmill walking [*This intervention has not been considered in the analysis*].	N/E	**HR**: HR increases with exercise prior to sitting compared to control trial. **BP**: positive effect of exercise prior to sitting compared to control trial.
Wheeler et al. [50]	Randomized cross-over trial	12	2 women 10 men	70 ± 7	30.4 ± 4.3	N/E	Resting BP and heart rate were measured by an automatic oscillometric device.	Participants completed 3 laboratory trial conditions. (1) uninterrupted sitting [8 h, control], (2) exercise prior to sitting: sitting [1 h], then moderate-intensity treadmill walking for 30 min followed by uninterrupted sitting [6.5 h] and (3) exercise + breaks: sitting [1 h], then moderate-intensity treadmill walking for 30 min followed by sitting [6.5 h] interrupted every 30 min with 3 min of light-intensity treadmill walking [*This intervention has not been considered in the analysis*].		**HR**: HR increases with exercise prior to sitting compared to control trial. **BP**: no effect of exercise prior to sitting compared to control trial.
Tucker et al. [47]	Randomized cross-over trial	13	13 men	27 ± 1	25.6 ± 1.1	Assessed with high-resolution ultrasonography on the brachial artery, which allowed the calculation of %FMD and SR.	N/E	Participants underwent three randomized conditions. (1) four 4-min intervals at 85-95% of maximum heart rate separated by 3 min of active recovery [HIIE 4 × 4], (2) 16 1-min intervals at 85–95% of maximum heart rate separated by 1 min of active recovery [HIIE 16 × 1], and (3) sedentary control. After the interventions, the patients remained sedentary for up to 18 h (the interventions and first assessment were performed between 1:00 p.m. and 2:00 p.m. The second assessment was performed in the morning).	**FMD**: positive effect of both exercise interventions compared to the control trial after sedentary behavior. **SR**_**AUC**_: positive effect of HIIE 4 × 4 compared to control trial after sedentary behavior.	N/E
Ballard et al. [30]	Randomized cross-over trial	11	11 men	21.2 ± 1.9	24.7 ± 1.0	Assessed with high-resolution ultrasonography on superficial femoral artery, which allowed the calculation of %FMD and SR.	BP was assessed in brachial artery using an automated blood pressure monitor.	Participants completed two 3 h sitting trials that were preceded by (1) 45 min of either quiet rest or (2) a 45 min single bout of continuous treadmill exercise [65% maximal oxygen consumption]	**FMD**: positive effect of exercise prior to sedentary behavior compared to control trial. **SR**: positive effect of exercise prior to sedentary behavior compared to control trial. **SR**_**AUC**_: No effect of exercise prior to sedentary behavior compared to control trial.	**BP**: no effect of exercise prior to sedentary behavior compared to control trial.
Morishima et al. [32]	Randomized cross-over trial	15	5 women 10 men	26.7 ± 0.5	25.6 ± 0.5	Assessed with high-resolution ultrasonography on the popliteal artery, which allowed the calculation of %FMD, SR and BF.	BP was assessed in brachial artery using an automated blood pressure monitor.	Participants completed three experimental trials. (1) sitting without prior exercise [Control Trial], (2) sitting with prior exercise [Subjects performed 45 min of leg cycling on a stationary bike. The workload was individualized for each subject to target a rate of perceived exertion of 11–13 (Borg Scale, 6–20)] and (3) standing without prior exercise [*This intervention has not been considered in the analysis*]. After 45 min of supine resting or cycling, subjects were positioned into a seated position, according to the trial, for 3 h.	**FMD**: positive effect of exercise prior to sedentary behavior compared to control trial. **SR**: positive effect of exercise prior to sedentary behavior compared to control trial. **SR**_**AUC**_: positive effect of exercise prior to sedentary behavior compared to control trial. **BF**: no effect of exercise prior to sedentary behavior compared to control trial.	**BP**: no effect of exercise prior to sedentary behavior compared to control trial.
Zhou et al. [51]	Randomized cross-over trial	19	19 men	24.7 ± 0.3	21.9 ± 0.3	N/E	Central vascular parameters at rest, BP and HR were measured with a device to measure vascular compliance (VaSera VS-1000 vascular screening system).	Participants completed 4 laboratory trial conditions. (1) no-exercise control [CON], (2) continuous exercise [CE, 30-min cycling], (3) accumulated exercise with 10-min intervals [AE10, 3 × 10-min cycling, 10-min interval], and (4) accumulated exercise with 60-min intervals [AE60, 3 × 10-min cycling, 60-min interval]. The exercise intensity for cycling in this study was set at 50% of heart rate reserve. After the interventions, the patients remained sedentary for 1 h.	N/E	**HR**: no effect of any intervention compared to control trial at 1 h **BP**: no effect of any intervention compared to control trial at 1 h
Younger et al. [52]	Randomized cross-over trial	10	4 women 6 men	22.2 ± 1.3	25 ± 2	N/E	BP was assessed with an auscultatory technique and an aneroid sphygmomanometer. HR was measured with a continuous pulsometer.	Participants completed two 5 h sitting trials that were preceded by (1) 45 min of either quiet rest or a (2) 30 min of moderate aerobic exercise.	N/E	**HR**: HR increases with exercise prior to sitting compared to control trial at 1 h and 2 h. No effect at 5 h. **BP**: no effect of exercise prior to sedentary behavior compared to control trial.

AE: Aerobic exercise; BF: Blood Flow; BP: Blood pressure; CE: Continuous exercise; CON: Control; FMD: Flow-mediated dilation; HIIIE: High-intensity interval exercise; HR: Heart rate; LV-HIIE: Low-Volume High-intensity interval exercise; SR: Shear rate; SR_AUC_: Shear rate area under the curve

### Effect of Exercise Prior to Sedentary Behavior on Vascular Function Parameters

#### FMD

All articles that assessed the effect of prior exercise on FMD showed that it was able to mitigate SB-induced endothelial dysfunction. This effect was observed both in cases where FMD was measured in an upper limb artery (brachial artery [37, 47]) and in a lower limb artery (superficial femoral artery [30] or popliteal artery [32]). The two studies that utilized 45-minute prior moderate-intensity aerobic exercise such as walking on a treadmill [30] or cycling [32] evaluated FMD in a lower limb artery and showed an increase in FMD up to the third hour of prolonged sitting compared to control condition (uninterrupted sitting without prior exercise). The other two studies that used high-intensity interval exercise as intervention prior to SB evaluated FMD in an upper limb artery. Of these studies, one demonstrated an increase in FMD following exposure to SB [47], while the other showed this effect only after 1 h of SB [37].

**Fig. 2:**
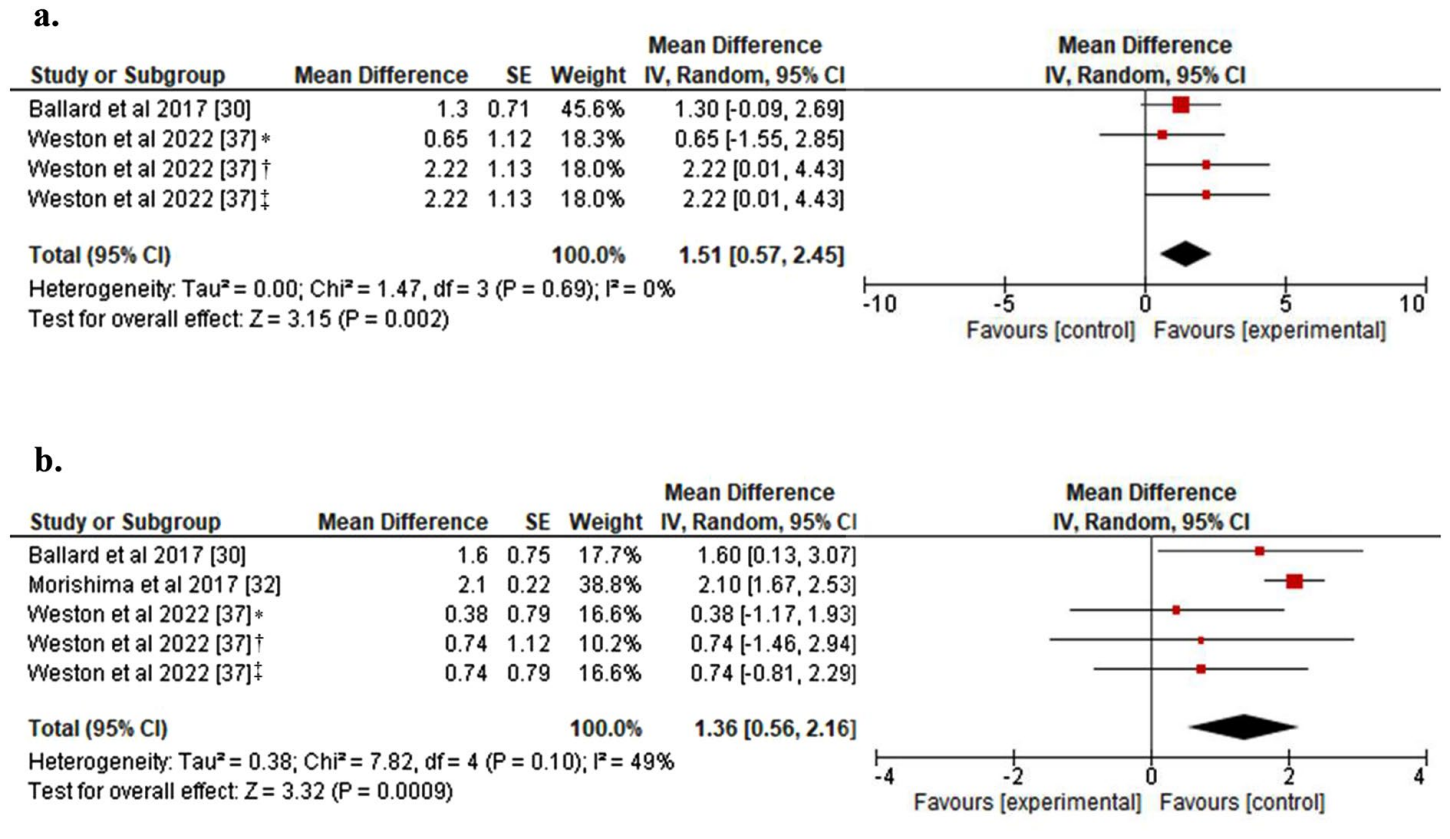
(**a**) Forest plot for the effect of exercise prior to SB on FMD at 1 h; (**b**) Forest plot for the effect of exercise prior to SB on FMD at 3 h. FMD: Flow-mediated dilation; SB: Sedentary behavior. *Moderate Intensity exercise; †HIIE1: 30 min of high intensity interval exercise at 75% VO2max; ‡HIIE2: 30 min of high intensity interval exercise at 90% VO2max

The results of the quantitative analysis (Fig. 2) showed that exercise performed prior to prolonged SB was able to mitigate the effect of sedentary behavior on endothelial function, represented by a greater %FMD compared to the control condition of SB without prior exercise. This effect was observed both at the first hour (MD: 1.51%; 95% CI: 0.57 to 2.45; *p* < 0.01; Fig. 2a) and at the third hour of sedentary time (MD: 1.36%; 95% CI: 0.56 to 2.16; *p* < 0.001; Fig. 2b). The analysis showed statistical homogeneity for the effect at the first hour (I^2^: 0%) and moderate heterogeneity for the effect at three hours (I^2^: 49%).

#### SR

Of the four studies that analyzed the effect of exercise prior to SB on SR, three reported an increase, while the fourth showed no effect. The two studies that measured SR in a leg artery evaluated 45 min prior exercise at moderate intensity (treadmill [30] and cycling [32]). These studies reported a higher resting mean SR compared to the control condition. In turn, the study conducted by Morishima et al. [32] also found that previous exercise had a protective effect on the hyperemic response (SR_AUC_). Studies that measured SR in an arm artery utilized prior high-intensity exercise. One of these studies reported a higher SR_AUC_ compared to the control [47], while the other showed no effect [37]. These studies did not measure the resting mean SR.

**Fig. 3:**
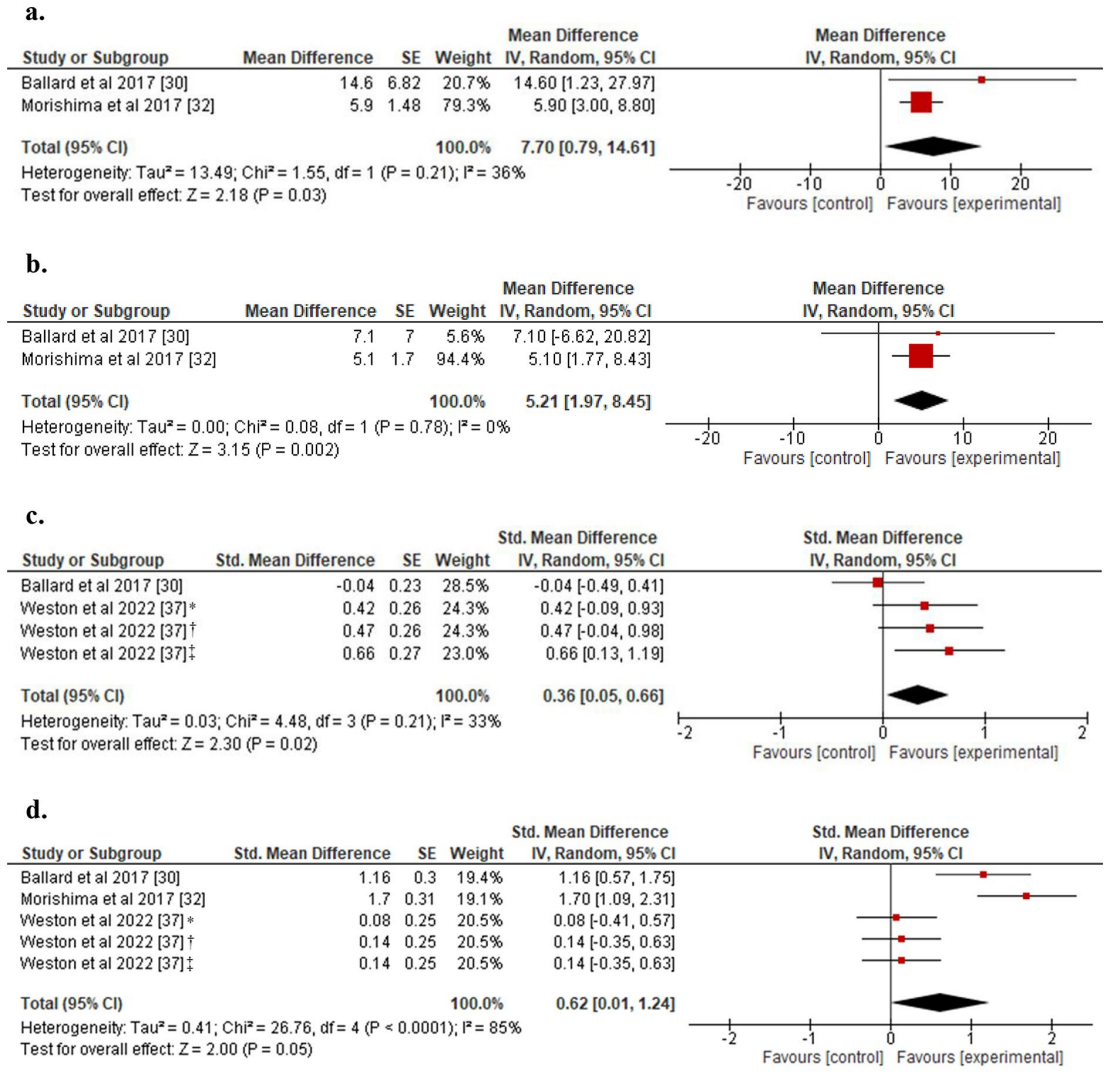
(**a**) Forest plot for the effect of exercise prior to SB on SR at 1 h; (**b**) Forest plot for the effect of exercise prior to SB on SR at 3 h; (**c**) Forest plot for the effect of exercise prior to SB on SR_AUC_ at 1 h; (**d**) Forest plot for the effect of exercise prior to SB on SR_AUC_ at 3 h. SB: Sedentary behavior; SR: Shear rate; SR_AUC_: Shear rate area under the curve *Moderate Intensity exercise; †HIIE1: 30 min of high intensity interval exercise at 75% VO2max; ‡HIIE2: 30 min of high intensity interval exercise at 90% VO2max

The meta-analytic findings revealed that prior exercise attenuated the detrimental effect of prolonged sedentary behavior on SR (Fig. 3), resulting in a higher SR compared to the control condition of SB without prior exercise, both in the first hour (MD: 7.70 s^^−1^; 95% CI: 0.79 to 14.61; *p* < 0.05; Fig. 3a) and in the third hour of sedentary time (MD: 5.21 s^^−1^; 95% CI: 1.77 to 8.43; *p* < 0.01; Fig. 3b). The statistical heterogeneity in the observed effect during the first hour was moderate (I^2^: 36%), while for the three hours following the exposure, there was statistical homogeneity (I^2^: 0%). Regarding the SR_AUC_ analysis, a small yet significant favorable effect of exercise prior to SB was observed towards the end of the first hour of sedentary time (SMD: 0.36; 95% CI: 0.05 to 0.66; *p* < 0.05; Fig. 3c) and a non-significant effect towards the end of the third hour (SMD: 0.62; 95% CI: 0.01 to 1.24; p: 0.05; Fig. 3d), compared to the control condition. The heterogeneity in the observed effect during the first hour was moderate (I^2^: 33%).

#### BF

Neither of the two studies that analyzed the effect of prior exercise on BF during prolonged sitting (a form of SB) showed a significant effect compared to the control condition (uninterrupted sitting without prior exercise).

**Fig. 4:**
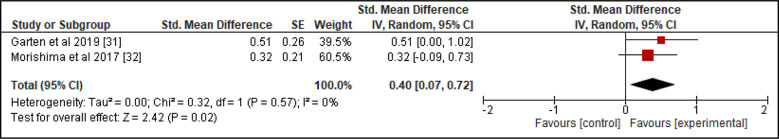
Forest plot for the effect of exercise prior to SB on BF at 3 h. BF: Blood Flow; SB: Sedentary behavior

The meta-analytic results for BF (Fig. 4) showed a small significant favorable effect of prior exercise towards the end of three hours of prolonged sitting, compared to the control condition (SMD: 0.40; 95% CI: 0.07 to 0.72; *p* < 0.05). There was no heterogeneity in the observed effect (I^2^: 0%). The analysis of the effect of prior exercise on BF could only be performed for the three hours of prolonged sitting, considering the available data in the included studies.

### Effect of Exercise Prior to Sedentary Behavior on Hemodynamic Parameters

#### BP

Regarding the studies that assessed the effect of prior exercise on BP during exposure to SB, three of them showed a reduction in mean BP [38, 48, 49] and seven reported no effect [30, 32, 37, 50–53]. The analysis of BP considered the mean arterial pressure obtained in the included studies during the experimental intervention (prior exercise to prolonged SB) and the control condition (prolonged SB without prior exercise).

**Fig. 5:**
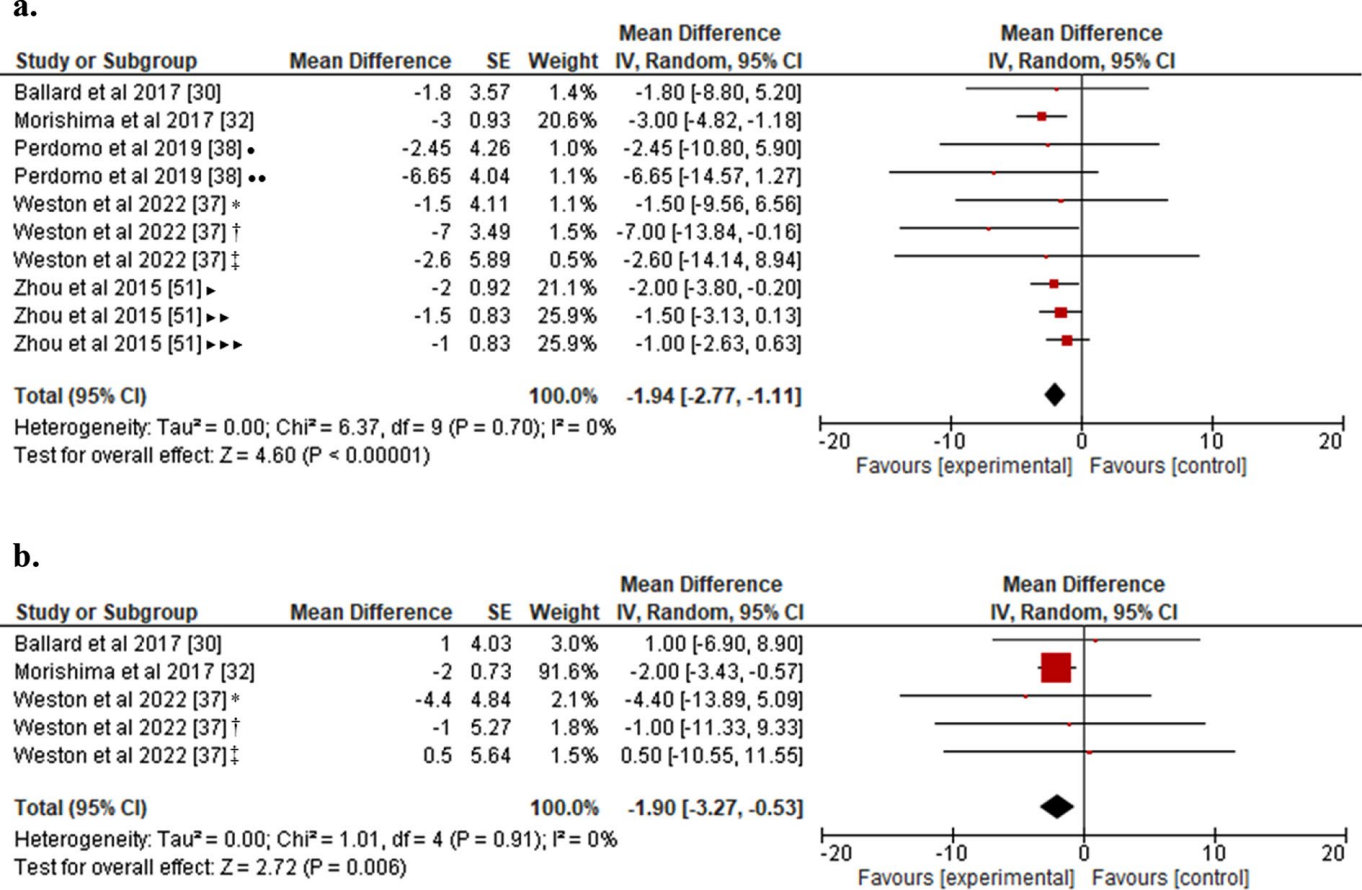
(**a**) Forest plot for the effect of exercise prior to SB on BP at 1 h; (**b**) Forest plot for the effect of exercise prior to SB on BP at 3 h. SB: Sedentary behavior; BP: Blood pressure. •10-min exercise bout; ••30-min exercise bout; *Moderate Intensity exercise; †HIIE1: 30 min of high intensity interval exercise at 75% VO2max; ‡HIIE2: 30 min of high intensity interval exercise at 90% VO2max; ‣Continuous exercise; ‣‣AE10: 3 × 10-minutes of cycling sessions with 10-minute breaks; ‣‣‣AE60: 3 × 10-minutes of cycling sessions with 60-minute breaks

The quantitative results (Fig. 5) showed that prior exercise to SB leads to a reduction in mean BP, both in the first hour (MD: -1.94 mmHg; 95% CI: -2.77 to -1.11; *p* < 0.0001; Fig. 5a) and in the third hour of prolonged SB (MD: -1.90 mmHg; 95% CI: -3.27 to -0.53; *p* < 0.01; Fig. 5b). The analysis showed no heterogeneity at both assessed time points (I^2^: 0%).

#### HR

Five out of six studies that assessed the effect of prior exercise on HR during SB reported an increase in HR [38, 48–50, 52], while one study showed no changes [51]. All of them assessed the effect after 1 h of SB.

**Fig. 6:**
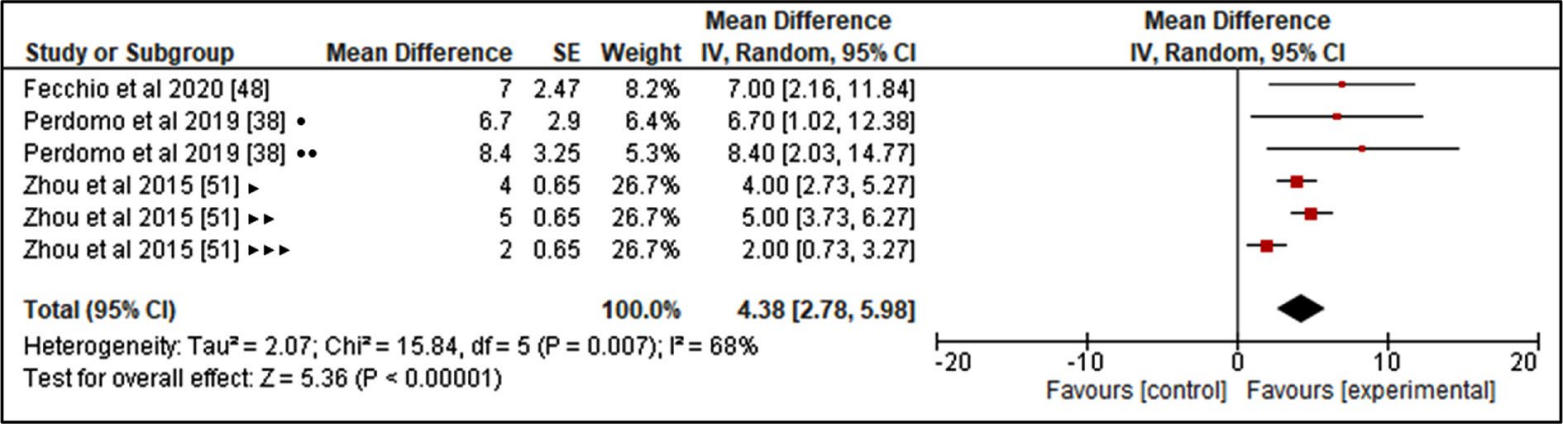
Forest plot for the effect of exercise prior to SB on HR at 1 h. HR: Heart rate; SB: Sedentary behavior •10-min exercise bout; ••30-min exercise bout; ‣Continuous exercise; ‣‣AE10: 3 × 10-minutes of cycling sessions with 10-minute breaks; ‣‣‣AE60: 3 × 10-minutes of cycling sessions with 60-minute breaks

The quantitative analysis results (Fig. 6) showed that exercise performed prior to SB leads to an increase in HR towards the conclusion of the first hour, compared to the control condition (MD: 4.38 beats per minute; 95% CI: 2.78 to 5.98; *p* < 0.0001). The heterogeneity for this analysis was moderate (I^2^: 68%). For this analysis of the effect of prior exercise on HR, it was only feasible to register assessments during the initial hour following SB exposure, based on the available data from the included studies.

### Sensitivity Analysis

A sensitivity analysis was performed using the sequential elimination method for all outcomes studied. For FMD and SR, it was observed that the results maintained their statistical significance even after the sequential elimination of articles from the meta-analysis. Regarding BF, it was found that the statistical significance was modified, but given the low risk of bias present in the articles, it was not considered appropriate to eliminate any of them from the analysis [54]. Similarly, BP and HR maintained their statistical significance in support of an effect of exercise prior to SB, through the sensitivity analysis.

### Risk of Bias and Quality of the Evidence

#### Risk of Bias

**Fig. 7:**
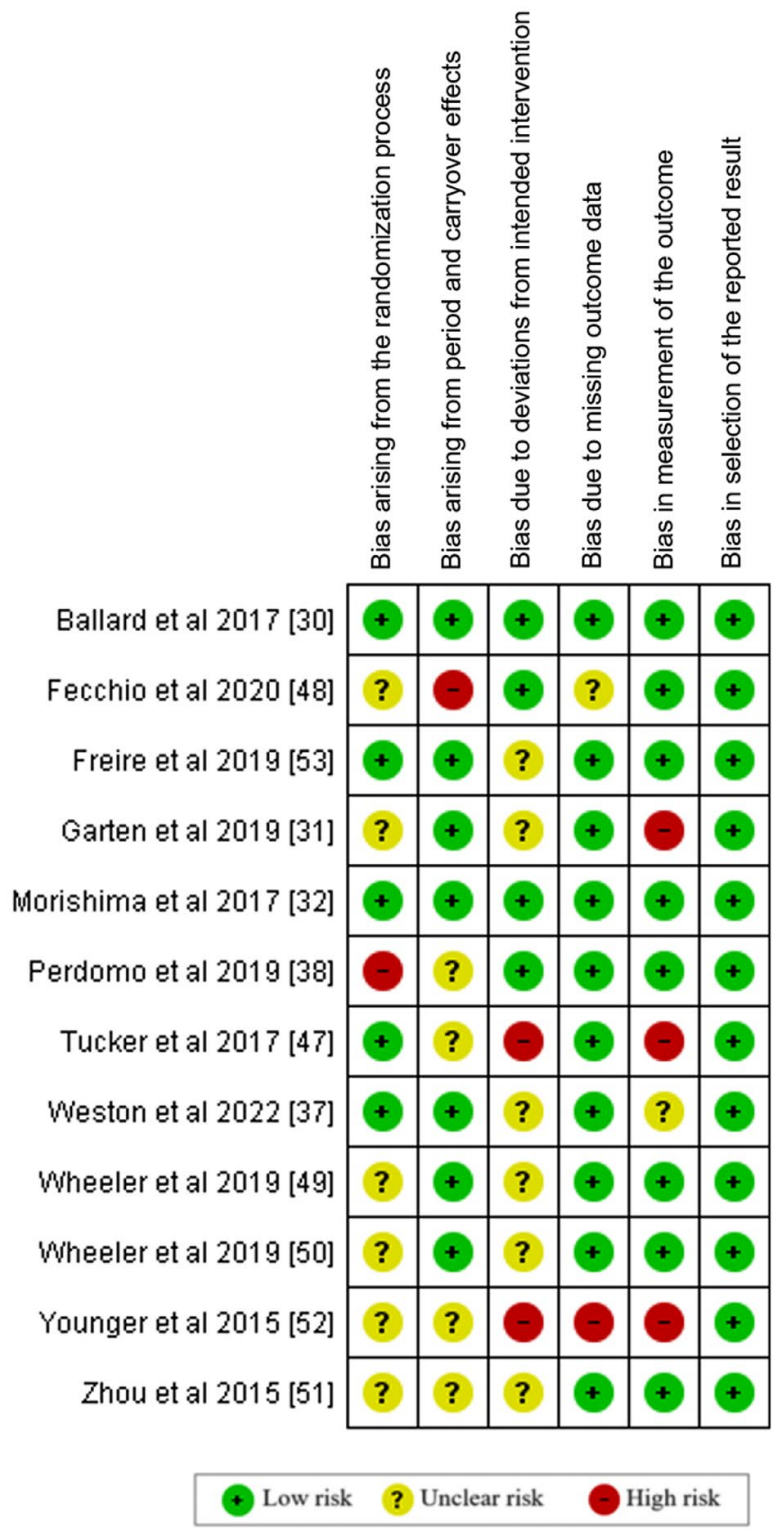
Summary of risk of bias for each primary study

**Fig. 8:**
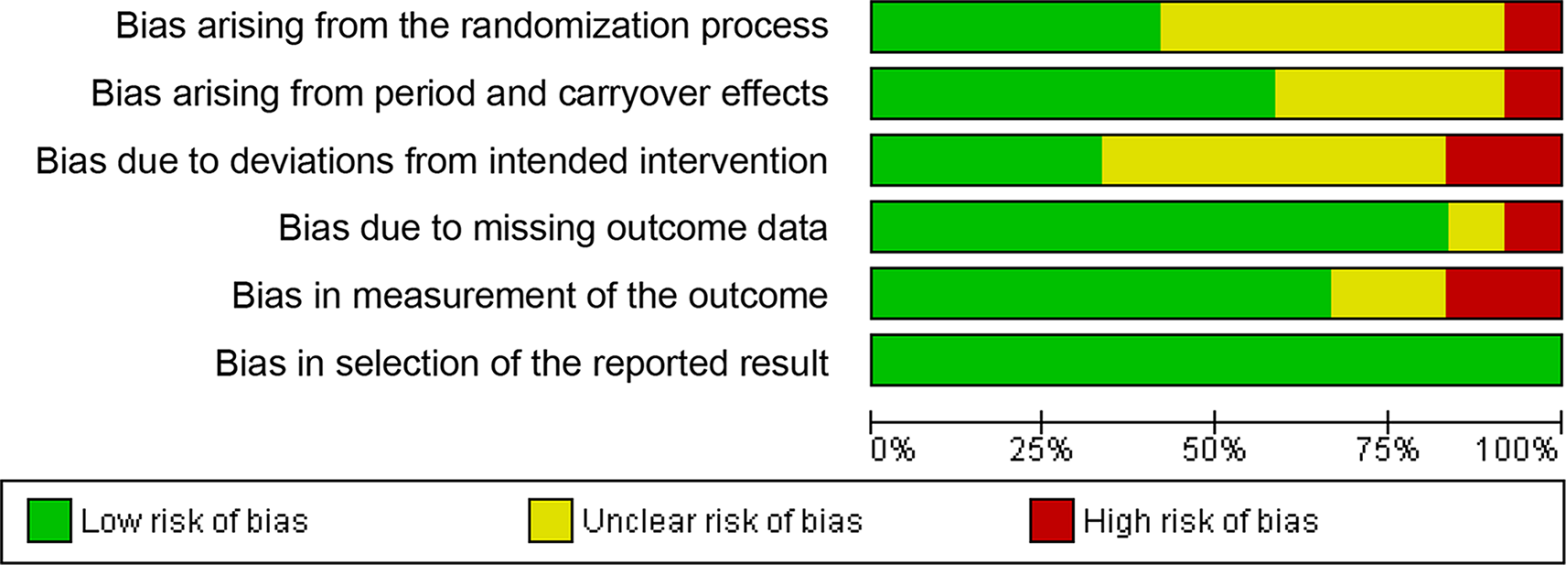
Risk of bias graph - review authors’ judgements about each risk of bias item presented as percentages across all included studies

A summary of the risk of bias for each study included is presented in Fig. 7. High risk of bias in the primary studies included in this meta-analysis arose from two main sources: bias in the measurement of the outcome (20%) and bias due to deviations from the intended intervention (20%). The presence of “some concerns” of risk of bias mainly derived from two sources: bias arising from the randomization process (50%), and bias due to deviations from the intended intervention (50%). Less frequently, bias can also arise from period and carryover effects (35%). The risk of bias graph is shown in Fig. 8.

#### Quality of the Evidence

The quality of evidence was classified as moderate for the outcome measures, except for SR, for which low quality was found. Initially, high quality of evidence was assumed for all evaluated outcomes; however, it was downgraded due to inconsistency caused by wide variation of point estimates (discrepancy or variability in results obtained from different studies) and substantial heterogeneity. Other reasons for downgrading the evidence included the presence of indirectness of evidence (absence of a direct correspondence between the data from available studies and the research question) and suspected publication bias (tendency to predominantly publish positive or significant results, while negative or non-significant results are less frequently published or remain unpublished). Table 2 summarizes the quality assessment process.

**Table 2:** Evidence quality assessment (certainty assessment)

Certainty assessment							Certainty	Importance	
No. of studies	Study design	Risk of bias	Inconsistency	Indirectness of evidence	Imprecision	Other considerations			
**FMD**									
4	Randomized trial	Not serious	Not serious	Serious ^a^	Not serious	None	⨁⨁⨁◯ Moderate	CRITICAL	
**SR**									
4	Randomized trial	Not serious	Serious ^b, c^	Serious ^a^	Not serious	None	⨁⨁◯◯ Low	IMPORTANT	
**BF**									
2	Randomized trial	Not serious	Not serious	Not serious	Not serious	Publication bias strongly suspected ^d^	⨁⨁⨁◯ Moderate	IMPORTANT	
**BP**									
10	Randomized trial	Not serious	Serious ^b^	Not serious	Not serious	None	⨁⨁⨁◯ Moderate	CRITICAL	
**HR**									
6	Randomized trial	Not serious	Serious ^c^	Not serious	Not serious	None	⨁⨁⨁◯ Moderate	IMPORTANT	

**FMD**: Flow mediated dilation; **SR**: Shear rate; **BF**: Blood flow; **HR**: Heart rate; **BP**: Blood pressure

^a^ The study by Tucker et al. [47] performed a different CS intervention than the other three studies that have evaluated this outcome

^b^ Wide variation in point estimates

^c^ Substantial statistical heterogeneity was found for this outcome

^d^ A limited number of articles have assessed this outcome. Possible existence of time bias

## Discussion

The main findings obtained were as follows: (1) Prior exercise may mitigate the impairment of SB-induced endothelial function, evidenced by a higher %FMD compared to the control condition at the first and third hours of sedentary time. Furthermore, a similar effect was observed on SR and BF. (2) Prior exercise also appears to impact central hemodynamic parameters, leading to a reduction in mean BP during the first and third hours of sedentary behavior. However, it causes an increase in HR during the first hour, compared to the control condition of SB without prior exercise. The average quality of the evidence that answers the research question of this study has been estimated as moderate. This fact implies that the findings could be transferred to possible clinical practice guidelines. However, the authors of this study recommend that further research be conducted in this area to reinforce the results obtained.

### Prior Exercise and Vascular Health Parameters

The results of this meta-analysis have shown that exercise performed prior to SB has a positive effect on %FMD. In both the first and third hours of continuous SB, there was an average increase in %FMD of 1.51% and 1.36%, respectively, compared to SB without prior exercise. This finding is clinically relevant, as an increase in %FMD is considered an important protective factor for the cardiovascular system, both in healthy individuals and those with cardiovascular pathologies [55]. In this regard, it has been reported that a 1% increase in FMD could reduce the risk of cardiovascular events by 8% [56] to 13% [13]. Several studies have highlighted the powerful effect that physical exercise has on endothelial function [57–60], showing an increase of up to 8.48% in FMD immediately after an aerobic exercise session [57]. This increase in FMD following exercise could be responsible for mitigating SB-induced endothelial dysfunction. According to the findings of this meta-analysis, its preventive effect could even persist up to the third hour of prolonged sitting time.

The results of this study can be compared to findings from other recent research assessing the effects of interrupting SB on endothelial function. In this line, studies such as Paterson et al. [33] and Soto-Rodríguez et al. [34] report that interrupting prolonged sitting with light or moderate intensity exercise strategies is associated with significantly better endothelial function compared to prolonged uninterrupted sitting. Although interrupting prolonged sitting is a different intervention than prior exercise, both strategies aim to prevent and maintain endothelial function.

This study has revealed that engaging in prior exercise before prolonged SB could have a beneficial and protective effect against the detrimental impact that SB has on SR. Given the importance of SR as a stimulator of nitric oxide production, its alteration is closely related to the onset of endothelial dysfunction [58, 61]. In this sense, it has been reported that SB produces a reduction in SR, decreasing %FMD [22, 62, 63]. Numerous studies have reported that regular physical exercise can counteract the negative effects of SB, either by interrupting it or by engaging in physical exercise prior to sedentary periods [30, 32–34, 36, 57, 64, 65]. A recent meta-analysis found that interrupting prolonged sitting with physical exercise is associated with a significant increase in SR compared to prolonged sitting without interruptions (MD: 7.58 s^^−1^; 95% CI: 3.00 to 12.17) [34]. Despite the differences in the physical exercise intervention described in that meta-analysis, its findings are similar to those seen in the present study, where prior exercise resulted in a comparable increase in SR (MD: 7.70 s^^−1^; 95% CI: 0.79 to 14.61). However, differences were observed in with the effect of prior exercise towards the end of the third hour of SB (MD: 5.21 s^^−1^; 95% CI: 1.77 to 8.43), although it remained significant. These results underscore the importance of physical exercise, in its various forms, for maintaining vascular health and preventing deterioration due to prolonged SB. Furthermore, they support the evidence of the relationship between the effect on SR and the response in %FMD [20].

This meta-analysis also found that exercise prior to SB might have a beneficial effect on BF. It is widely accepted that preserving BF is highly relevant to maintaining vascular health. Changes in this factor have effects on vasomotor endothelial function since they directly modify the stimulus caused by SS (and its respective SR) [66]. Sitting and the consequent “arterial bending” have been reported to reduce blood flow due to increased resistance and obstruction of normal BF [32, 67]. Several studies have reinforced this idea, finding significant reductions in BF after prolonged periods of SB [19, 22, 68]. In contrast, physical exercise has been shown to improve BF significantly and, therefore, may constitute a relevant preventive intervention strategy for preserving BF [69]. Cho et al. [24] found that the interruption of prolonged sitting with physical exercise resulted in increased BF compared to a control condition (uninterrupted sitting). The results of this meta-analysis are consistent with these reports, as it was found that exercise prior to prolonged sitting produces significantly higher BF even after three hours of uninterrupted sitting. These findings further reinforce the assertion that exercise serves as a preventive and therapeutic strategy for alterations in endothelial function induced by SB.

Regarding the described effects, these seem to be consistent with those found in the literature, where it has been reported that all intensities of exercise significantly improve endothelial function [58, 59, 70]. However, the findings of this study did not support the presence of a dose-response relationship between exercise intensity and endothelial function. [70]. This discrepancy could be explained by three fundamental reasons; (1) the variation in the arteries where the measurement of endothelial function was performed, (2) the risk of bias of studies that employed high-intensity interval exercise versus moderate exercise, (3) the possible existence of a threshold effect in relation to the intensity of exercise prior to SB. Regarding the first reason, several studies have reported that the influence of SB on endothelial function may vary according to the type of artery [22, 25, 71]. This variability can be attributed to differences in the levels of NO present in various arteries [72], which may result in varying responses depending on the modality, intensity, and duration of the prior exercise. Moreover, the femoropopliteal arterial segment, due to its exposure to bending and traction forces, is particularly susceptible to the negative consequences of sitting position [73] or maintained supine position [74]. On the other hand, methodological bias and the limited number of existing studies could be reducing the statistical power for detecting the effect or underestimating the effect of those studies that employed high-intensity exercise in comparison to moderate-intensity exercise. Finally, the existence of a threshold effect could explain why a higher intensity of prior exercise does not result in greater benefits after a prolonged period of SB. In other words, despite the immediate intensity-dependent effects of exercise on endothelial function parameters, the subsequent “residual” effects behave similarly over time during SB exposure. However, further studies are required to analyze the effects of different intensities of prior exercise and explore the dose-response curve after prolonged SB exposure.

### Prior Exercise and Hemodynamic Parameters: BP and HR

Regarding mean BP, this study has found that prior exercise to SB exposure produces a similar reduction with a small effect size, both at one hour and three hours. These findings are in line with those reported in a recent meta-analysis, which determined that interrupting prolonged sitting through exercise produces small reductions in BP [64]. It has been shown that reducing BP is associated with a lower risk of cardiovascular diseases [75, 76], therefore, its modification has high preventive relevance. While the results of this study only provide an approximation of the changes that occur during an acute session, repeating these exercise patterns could result in a sustained reduction in BP [59], thus allowing a reduction of the cardiovascular risk derived from prolonged exposure to SB.

This meta-analysis has shown that exercise prior to SB exposure increases HR by approximately 4 beats per minute following 1 h of SB. However, the studies analyzed did not measure HR after a longer period of SB exposure (i.e., over 1 h). Although the increase in HR found in this study is not clinically relevant, it may reflect a greater demand for BF by the tissues after exercise. It has been shown that variations in BF are directly related to SS and, therefore, to endothelial NO production [61]. While previous studies have reported an elevation in HR following prolonged SB exposure [28, 29], this phenomenon might serve as a compensatory mechanism to sustain BF and, thus, SS. Therefore, the increase in HR induced by prior exercise could potentially enhance this mechanism. Nonetheless, further experimental studies are warranted to elucidate these mechanisms more comprehensively.

### Limitations

Firstly, due to the number of studies included in this meta-analysis, it was not possible to conduct a subgroup analysis to explore potential differences based on sex, exercise intensity, and arterial regions. Secondly, the follow-up measurements carried out for the SR variable at three hours presented a statistical heterogeneity greater than 70%. Therefore, this aspect should be considered in the interpretation of this result by readers. Finally, some studies could not be included in the estimation of effect size in some forest plots due to the lack of sufficient data in the published primary studies, or because no response was obtained from the researchers.

## Conclusions

The practice of exercise prior to SB appears to mitigate the impairment of vascular function and central hemodynamic parameters caused by prolonged sessions of SB. While favorable results have been observed for these exercise strategies, the quality of evidence was estimated as moderate. Therefore, further experimental research and high-quality clinical trials are needed in this area to strengthen the results and conclusions drawn.

## Data Availability

The data that support the results have been extracted from the sources and databases described in this research. However, the data extraction tables are available to be sent at the request of those who require it.

## Abbreviations

AE: Aerobic exercise
BF: Blood Flow
BP: Blood pressure
FMD: Flow-mediated dilation
HIIE: High-intensity interval exercise
HR: Heart rate
LV-HIIE: Low-Volume High-intensity interval exercise
SR: Shear rate
SR_AUC_: Shear rate area under the curve
SS: Shear stress
